# The Treatment of Abscesses by Hyper-Dentition with Carbolized Water

**Published:** 1877-01

**Authors:** George W. Callender

**Affiliations:** Surgeon to St. Bartholomew's Hospital.


### ARTICLE VII.

### *The Treatment of Abscesses by Hyper-Distention with Carbolized Water.*

By George W. Callender, F. R. S., Surgeon to St. Bartholomew's Hospital.

In the treatment of all abscesses, but more especially for those with branching sinues, or with a sac broken up by imperfect septa, we have found it desirable not merely to wash out the abscess in what, for distinction, may be termed the ordinary way, but to throw in such a quantity of fluid as will distend the abscess sac in all its parts; and this produce we speak of as hyper-distention of an abscess cavity. In this manner, abscesses complicated in the way I have mentioned, may be cured as effectually as are those in which we have to deal with a single cavity.

The operation may be performed whilst the patient is under the influence of ether, or the integuments may be frozen by the ether spray. The following are required:—A scalpel where an incision is needed, no open sinus existing; carbolic acid lotion (one part in twenty) diluted to one in thirty by the addition of warm water before using it; a perforated elastic drainage-tube; carbolized oil (one in twelve) on lint for dressing the wound, and gutta-percha tissue for covering this; some ordinary adhesive plaster; some tenax to receive any subsequent discharge (which, however, is very slight;) an ordinary two or four-ounce syringe. When it is desirable to make continuous pressure over an abscess after opening it, a pad shaped to the needs of the case, and filled with shot, will be found useful. It acts more affectually than a sand-bag, and is easily made and adapted.

The operation is begun by cutting into the abscess (if no sinus exist), the opening made being of sufficient size to admit one of the fingers. The pus is then allowed to escape, the abscess being emptied as completely as possible. The nozzle of a syringe is next passed through the opening, and the skin is drawn closely around it by the operator with his left hand; the contents of the syringe are then passed into the abscess sac. Care must be taken, in doing this, that no pressure is made upon the abscess wall, or the distention of the sac will be incomplete. Either by using a syringe which throws a continuous stream, or equally well by closing the wound with a finger whilst the syringe is being refilled by an assistant (very little fluid being lost in its reintroduction), the abscess sac will presently distend quite to, or even beyond, its original size; and, under these circumstances, the carbolized water necessarily finds its way (as a rule, which has few exceptions) into all parts of the cavity, however irregular, and along any channels leading from it.

When the abcess has been opened, the amount of injection may be roughly measured as being rather in excess of the quantity of pus let out. When distention has been effected, the fluid is allowed to escape, and, if much pus be mingled with it, a second injection may be practiced. An elastic drainage-tube, its size varying with that of the abscess, is then inserted and secured, and over the end of this, and over the wound, a piece of lint, twice folded and soked in carbolized oil, is laid. This is covered with a sheet of gutta-percha tissue and some tenax, and these dressings are secured with some ordinary plaster.

Subsequent treatment consists in the renewal of the dressings, which, to myself, it seems desirable to see to daily The drainage tube is gradually shortened as the abcess wall contracts, and through its canal, if there be any sign of puriform discharge, a little carbolized water may be occasionally injected.—*British Medical Journal.*

---

